# Special Issue “Aquaporins in Brain Disease”

**DOI:** 10.3390/ijms25063513

**Published:** 2024-03-20

**Authors:** Miriam Echevarría, Pablo García-Miranda

**Affiliations:** 1 Instituto de Biomedicina de Sevilla, IBIS/Hospital Universitario Virgen del Rocío/CSIC/Universidad de Sevilla, 41013 Sevilla, Spain; 2 Departmento de Fisiología Médica y Biofisíca, Facultad de Medicina, Universidad de Sevilla, 41009 Sevilla, Spain; 3 Departamento de Fisiología, Facultad de Farmacia, Universidad de Sevilla, 41012 Sevilla, Spain

Water is an abundant and important component of the human brain, the homeostasis of which is rigorously controlled. Aquaporins (AQPs) play a key role in mediating most water movement that occurs between different liquid-filled compartments of the human body: intracellular fluid (ICF), interstitial fluid (ISF), cerebrospinal fluid (CSF), and blood. The movement of water between the barriers that separate them is driven by osmotic forces and hydrostatic pressure gradients. Thus far, nine AQPs have been identified at different sites in the central nervous system (CNS), including AQP1, 3, 4, 5, 6, 7, 8, 9, and 11 [1]. Their functioning, particularly for AQP4 and AQP1 which are most abundant, has been associated with the production and drainage of cerebrospinal fluid (CSF) and the facilitation of water flow through brain compartments. Their potential involvement in the pathophysiology of many brain disorders has increased progressively and to date, they have been implicated in edema and congenital hydrocephalus, as well as idiopathic normal-pressure hydrocephalus, idiopathic intracranial hypertension, optic neuromyelitis, and neurodegenerative diseases such as Parkinson’s disease (PD) and Alzheimer’s disease (AD). Interestingly, AQP4 has recently been discovered to have a role as a protein marker for astrocytes and the astrocyte development stage. The presence of AQP4 is associated with the appearance of astrocytes in the brain, which in turn appear to contribute to postnatal angiogenesis, the formation of the blood–brain barrier, and even myelinization. 

“Aquaporins in Brain Disease I” comprises eight interesting articles addressing different aspects by which members of the aquaporin family expressed in the central nervous system (CNS) have been associated with neurodevelopment and neurological diseases, and in some cases, experimentation in animal models used to relate AQPs to brain physiology and pathology. 

The accumulation of misfolded or waste proteins is coming in some neurodegenerative disease, such as beta-amyloid and tau in AD or synuclein in PD. The glymphatic system plays a critical role in the clearance of protein depositing in the interstitial space of the brain, and AQP4 has been established as a central element for fluid transit between perivascular spaces that allow for an adequate transit of liquid and substances to the exit pathways of the brain. As detailed in the review by Yamada, C. [2], the crucial roles of AQP4 in Alzheimer’s disease pathogenesis are strongly supported by experiments conducted in animal models lacking AQP4, in which a clear exacerbation of the pathology of AD was observed. Furthermore, most studies indicate that glymphatic clearance is suppressed in AD patients, but the exact mechanisms by which the regulation of AQP4 expression contributes to suppression of the glymphatic system require further exploration. Other factors independent of AQP4 can also influence the glymphatic flow, for example, extracellular tortuosity of the extracellular space or interactions of solutes with elements of the extracellular matrix, and contribute to reducing the glymphatic flow and promoting the onset of AD symptoms, which would require a better understanding of the process.

Consistent with this, the disruption of circadian rhythms has also been indicated among the different pathological symptoms of AD. The work of Carrero et al. [3] demonstrates an important reduction in the expression of AQP1, AQP4, and AQP5 in the retinal tissue of APP/PS1 mice, an animal model of Alzheimer’s disease. In these animals, the accumulation of amyloid B (AB) was also observed, which directly interferes with light information projected from the retina to the suprachiasmatic nucleus in the hypothalamus, thus affecting the regulation of the circadian rhythm. Therefore, the findings of these authors suggest that an abnormal transport of AB, mediated by impaired AQP expression contributes to retinal degeneration during the early stages of AD, eventually leading to changes in clock gene expression and the down-regulation of circadian rhythms. 

Bonosi, L. et al. [4] highlight the roles of AQPs in mediating certain pathological processes related to the genesis of epilepsy. Therefore, although epilepsy is a pathology mainly associated with impaired neuronal transmission, many accumulated studies have demonstrated the important role that astrocytes and, particularly, the expression of AQP4 on their membrane, have in the hydro-electrolyte balance necessary for neuronal activity. The expression of AQP4 in astrocyte endfeet, together with Kir4.1 K^+^ channels, is responsible for potassium homeostasis during neuronal activity. The malfunctioning of AQP4 may lead to an accumulation of extracellular K^+^, favoring a hyperexcitable state similar to that observed in epileptic brains, and will eventually lead to an increased susceptibility to seizures. Additionally, the dysregulation of glutamate transporters (GLT1 and mGluR) has also been associated with an altered expression of AQP4, also leading to extracellular glutamate accumulation, which, together with higher K^+^, creates a state of neuronal hyperexcitability clearly associated with epileptogenesis. 

The review presented by Toader, C. et al. [5] exhaustively describes the properties of the most relevant brain AQPs, comprehensively exploring how AQP1 and AQP4 contribute to water homeostasis in the brain. Apart from outlining their role in the glymphatic system and neurodegenerative disorders such as Parkinson’s and Alzheimer’s diseases, they also expose the relationship between these proteins and stroke, edema, and autoimmune pathological conditions such as neuromyelitis optica. However, their article scrutinizes the role of AQPs in tumorigenesis, cell migration, invasiveness, and angiogenesis. Furthermore, these authors discuss emerging aquaporin-targeted therapies that may have some interest in the future combating strategies for CNS malignancies and neurodegenerative disorders.

In the work of González-Marrero et al. [6], the authors analyze adaptive changes in the expression pattern of AQP1 and AQP4 in the brain of spontaneously hypertensive rats (SHR). These animals constitute the most commonly used animal model of hypertension, which is curiously also characterized by secondary ventricular dilation and hydrocephalus. In SHR, the expression of AQP1 and AQP4 decreased over time in places involved with CSF production, such as the choroid plexus and ependyma, while the levels of AQPs in the CSF increased. Thus, AQP alterations seem to obey to a compensatory mechanism by which production of the CSF is reduced via elimination of producer proteins (AQPs) from CSF secretory tissues and packaging them into vesicles that are extruded and floating into the CSF. As such, AQPs could potentially compensate for the hydrocephalus and would simultaneously serve as the CSF markers of such a process.

In the remaining three papers of this Special Issue, the presence of AQP4 in astrocytes is associated with gliogenesis and the supportive role that astrocytes offer to processes such as neural development, neurotransmission, blood–brain barrier formation, and myelinization. In different ways, the authors present evidence that associates the expression of AQP4 with the different stages of astrocyte maturation. Castañeyra-Ruiz et al. [7] revealed interesting data, proposing AQP4 as a possible biomarker for gliogenesis and ways in which alterations in AQP4 expression can be associated with pediatric hydrocephalus, with a specific emphasis on the repercussion of reactive astrogliosis in this pathological condition. According to Castañeira-Ruiz [7], unpolarized expression of AQP4 is found in proliferative cells with different morphologies in the human brain: in glial stem cells with long projections that are used for other cells to migrate and populate white matter tracks, and intermediate progenitor cells with an oval shape and lack of long projection. From these early stages of astrogenesis, AQP4 is expressed in polarized astrocyte endfeet, indicating the final stage of astrocyte maturation. Interestingly, a significant coincidence of this pattern of expression for AQP4 is also reported in neonatal mice by Mayo et al. [8], outline in this Special Issue. The observations of these authors depicted a clear switch in the expression of AQP4 from white- to gray-matter regions during postnatal development of the CNS. During the first week of life, the animals showed AQP4 labeling on exclusively less mature glial cells (large and with an unpolarized distribution of AQP4) in the corpus callosum, cerebellum, and spinal cord. On the other hand, in adult mice, AQP4 expression was concentrated in more mature astrocytes and mainly polarized at the endfeet–vessel interface in the tissues previously indicated. These findings allow the authors to suggest the possible role of AQP4 in the early cell differentiation process during the first days of life in the newborn animal, which may be important for myelinization. Furthermore, as indicated by Castañeira-Ruiz et al. in their review [7], in different animal models that end up developing congenital or perinatal hydrocephalus, changes in AQP4 expression cause premature differentiation of glial stem cells (GSC) into reactive astrocytes, causing neurodevelopmental disorders. This occurs because GSCs lose their projections toward the white matter tracks and glioblasts then lose their migration guidance for the correct formation of white matter, a typical finding observed in pediatric hydrocephalus. Partial agenesis or hypoplasia of the corpus callosum is seen in patients with spina bifida, cases of obstructive hydrocephalus, and often in patients with a posthemorrhagic hydrocephalus [7]. Finally, in line with the relevant role of AQP4 in congenital hydrocephalus, the work of García-Bonilla et al. [9] showed evidence that the injection of bone marrow-derived mesenchymal stem cells (BM-MSC) into the lateral ventricles of hydrocephalic mice (hyh) produced an astrocyte reaction that elicited an important overexpression of AQP4, together with induction of other neuronal process, and an overexpression of nerve growth factor (NGF) and vascular endothelia growth factor (VEGF), in addition to other factors that lead to recovery of the hydrocephalus condition. In conclusion, the role of AQPs in CNS physiology and pathophysiology is significant; nevertheless, new knowledge continues to emerge, making this a constantly progressing field.
